# Ownership and Utilisation of Long-Lasting Insecticidal Nets in Tiko Health District, Southwest Region, Cameroon: A Cross-Sectional Study

**DOI:** 10.1155/2021/8848091

**Published:** 2021-02-02

**Authors:** Paulette Ngum Fru, Frederick Nchang Cho, Andrew N. Tassang, Celestina Neh Fru, Peter Nde Fon, Albert Same Ekobo

**Affiliations:** ^1^Department of Public Health and Hygiene, Faculty of Health Sciences, University of Buea, P.O. Box 63 Buea, Cameroon; ^2^District Health Service Tiko, South West Regional Delegation of Health, Ministry of Public Health, Cameroon; ^3^Department of Biochemistry and Molecular Biology, Faculty of Science, University of Buea, P.O. Box 63 Buea, Cameroon; ^4^Catholic School of Health Sciences, Saint Elizabeth Hospital Complex, P.O. Box 8 Shisong-Nso, Cameroon; ^5^Central African Network for Tuberculosis, HIV/AIDS and Malaria (CANTAM), University of Buea, P.O. Box 63 Buea, Cameroon; ^6^Global Health Systems Solutions, Cameroon; ^7^Department of Obstetrics and Gynaecology, Faculty of Health Sciences, University of Buea, P.O. Box 63, Buea, Cameroon; ^8^Buea Regional Hospital Annex, Buea, Cameroon; ^9^Atlantic Medical Foundation, Mutengene, Cameroon; ^10^Department of Sociology and Anthropology, Faculty of Social and Management Sciences, University of Buea, P.O. Box 63 Buea, Cameroon; ^11^Solidarity Hospital, Buea, Cameroon; ^12^Faculty of Sciences, University of Yaoundé I, P.O. Box 337, Yaoundé, Cameroon; ^13^Faculty of Medicine and Pharmaceutical Sciences, University of Douala, Cameroon

## Abstract

**Introduction:**

Malaria is and remains a serious health concern in Africa. In Cameroon, where malaria is endemic and a major public health problem, the major control measure put in place is the use of long-lasting insecticidal nets (LLINs). In the Tiko Health District (THD), the challenges have been to assess and to evaluate the ownership and utilisation of LLINs. This study sought to assess the ownership and utilisation rates of LLINs in the THD. *Methodology*. A cross-sectional survey involving 418 households was conducted in four health areas in the THD. A structured questionnaire was used to collect data on LLIN ownership and utilisation as well as sociodemographic characteristics.

**Results:**

The ownership of at least one LLIN per household, coverage, and accessibility were, respectively, 89%, 56.2%, and 66.3%, while installing LLINs on all beds in the household, sleeping under LLINs the previous night (SULPN), and universal utilisation were 72%, 24.9%, and 14.1%, respectively. Factors significantly associated with the ownership of at least one LLIN per household were respondent's age and gender. Heat (21.1%) and forgetfulness (6.5%) were the main reasons postulated for irregular utilisation of LLINs.

**Conclusion:**

The ownership LLINs failed to guarantee utilisation and definitely effective control of malaria in the THD, as expected. Continuous and appropriate use of LLINs is indispensable, in addition to periodic sanitation, booster campaigns of LLIN distribution, and evaluation research for effective prevention and control of malaria.

## 1. Introduction

Malaria remains one of the greatest killer and devastating disease in Africa, a big threat to public health and economic burden despite all control strategies put in force by the National Malaria Control Programme, Global Fund for Health, Roll Back Malaria (RBM), and the World Health Organisation (WHO) [1–8]. In 2019, about 215 million cases, up from 214 million cases of malaria in 2014 [6], were reported leading to 384,000 deaths down from 438,000 deaths in 2015 [9, 10], about 94% of which occurred in the African region [7, 11].

In Cameroon, malaria morbidity and mortality have gone upwards since 2017 [11]; it is responsible for 30–35% of total annual death cases, accounting for 35% of childhood mortality and 40–45% morbidity [12]. Over 90% of Cameroonians are at risk of malaria infection, with an estimated 41% records of at least one episode annually, with pregnant women and children less than five years usually more vulnerable [12–17]. In the Southwest Region, 56% of hospital consultations, 54% of hospital admissions, and 53% of deaths among children below five years are due to malaria. Similarly, 42%, 70%, and 12% of hospital consultations, hospital admissions, and deaths among pregnant women are due to malaria [16, 18, 19].

The WHO's prevention package for the fight against malaria consists of vector control measures and preventive treatment strategies for the most vulnerable groups [1, 4–6, 10], of which vector control is the main approach to malaria prevention. Two forms of vector control (insecticide-treated mosquito nets (ITNs) and indoor residual spraying of insecticides) are effective in a wide range of circumstances [4, 10, 20, 21]. Recently, the scale-up of effective prevention tools has had a major impact in the fight against malaria. Thus, increased investment in proven prevention measures and in the development and deployment of new tools will accelerate progress towards a world free from malaria [6, 22]. In Cameroon, the mass distribution campaign (MDC) of long-lasting insecticidal nets (LLINs) was implemented in 2011, with about 8,654,731 LLINs distributed throughout the country [23]. This was followed by a second mass distribution in 2015 and a third with the distribution of about eight million LLINs in 2019 [7]. From 2011, ITN/LLIN ownership, coverage, and access have been on the increase [11] in some parts of the country. Unfortunately, very few studies have been carried out to routinely monitor and evaluate the ownership and utilisation of LLINs in Cameroon as a whole and in the Tiko Health District (THD) in particular [1, 2, 4, 12, 16, 18, 23–26]. Similarly, there has been no follow-up on the ownership and utilisation of LLINs in the THD after the 2011 and 2015 MDCs. To monitor the ownership and utilisation of LLINs, some LLIN indicators have to be considered: ownership of at least one LLIN per household, universal coverage, accessibility, use of LLINs last night, and universal utilisation. Such information is useful to determine the frequency of health education in order to enhance malaria prevention as the third MDC is yet to reach the study area due to the ongoing conflict in the region. This is more important as many inhabitants have exposed themselves to the malaria vectors, as internally displaced persons. The aim of this study was to assess the ownership and utilisation of LLINs in the THD.

## 2. Methods and Materials

### 2.1. Free Mass Distribution Campaign

The Cameroonian Ministry of Public Health undertook a nationwide free LLIN distribution campaign from health facilities to all households in the country at the end of 2011, with the objective to provide an insecticide-treated net (ITN), with a lifespan of five years, to all household beds or a LLIN for every two individuals per household, to a maximum of three ITNs per household, as described elsewhere [23, 26].

### 2.2. Study Design and Setting

This was a cross-sectional study conducted in June and July 2017 among household heads in four health areas in the THD. The THD (N 04°04′32.6^″^ E 009°21′28.9^″^) [23] is one of the 18 health districts in the Southwest Region of Cameroon. The health district has a population of about 334,647 people (mainly farmers and traders) distributed in eight health areas and covers a land surface of 484 km^2^ [27, 28]. Household heads or their representatives signed informed consents prior to filling the 27-item pretested questionnaires (S1 file: questionnaire). A structured household self-reporting questionnaire was designed to take about 15 minutes to administer and covered identification (health area and quarter of residence), ownership and sources of LLINs, utilisation of LLINs, and demographics of household heads. Household heads eligible to participate in the study were those who had lived in the household for at least one year; could speak Pidgin, English, or French; and were willing to give consent.

### 2.3. Sample Size Determination and Sampling

A minimum sample size of 384 was calculated with the CDC Epi Info version 7.2.2.6 (Centre for Disease Control, Georgia, USA) StatCalc with the following characteristics: an average population of 307,620 in 2009 with an annual increase rate of 2% (6152.4) to 369,144 in 2018 [29], estimated proportion of households owning LLINs of 50%, accepted error margin of 5%, design effect of 1.0, and one cluster.

We used the multistage cluster sampling method where a list of all the eight health areas, quarters therein, and the number of households were collected from the THD Service. A total of 20 quarters were selected, including at least three from each cluster. At least 31 households were selected from each health area, resulting in a total sample of 418 households. The sampling procedure of the required number of households was done in two stages.

#### 2.3.1. Stage One

**Figure 1:**
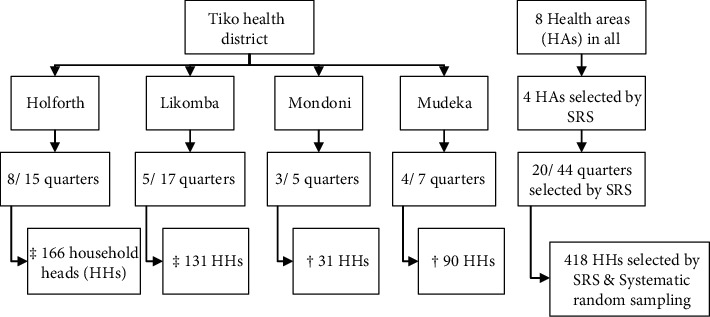
Multistage sampling. SRS: simple random sampling; HHs: household heads. ^‡^HHs sampled by systematic random sampling, ^†^HHs sampled by SRS.

We obtained household registration codes from previous MDCs and four clusters (HAs): Holforth, Likomba, Mondoni, and Mudeka were selected using simple random sampling (SRS) with probability proportionate to size (Figure 1). This was followed by listing and compiling all the number of households for each selected HA, and the required number of quarters was selected by SRS.

#### 2.3.2. Stage Two

Within each selected HA, households were selected as follows: for small quarters (less than 150 households), the entire quarter was mapped, and from the compiled list, households were selected by SRS. For quarters with more than 200 households, the systematic random sampling approach was used. From the main entrance of each quarter, every third or fourth house was sampled depending on the number of households in the quarter (Figure 1).

### 2.4. Concept Definitions

*A household* was defined as a wife with her direct dependents, and a compound was divided into several households depending on the number of wives, where the husband was assigned to the first wife's household [16, 30].

*Household ownership of LLINs* was defined as the proportion of households with at least one LLIN, where the numerator comprises the number of households surveyed with at least one LLIN and the denominator is the total number of households surveyed [5, 31–33].

*Coverage* was the proportion of households with at least a LLIN for every two persons, where the numerator comprises all households where the ratio between the number of LLINs owned and the number of *de jure* members of that household, that is, usual members excluding visitors, is 0.5 or higher and the denominator is the total number of sampled households [31–33].

*Accessibility* was the proportion of the population with access to LLINs in their households where the numerator includes all *de facto* household members in the sample who had access to a LLIN assuming each LLIN was used by two people and the denominator is the *de facto* population in the sample [32, 33].

*Household universal LLIN utilisation* is the proportion of population that slept under a mosquito net the previous night [31, 32].

*Slept under LLINs the previous night* (SULPN) is the proportion of household heads that slept under a mosquito net the previous night, where the numerator comprises the number of household heads who used LLINs last night and the denominator is the total number of households surveyed [34].

### 2.5. Data Analysis

We entered data into Epi Info version 7.2.2.6 (Centre for Disease Control, Georgia, USA) and analysed with IBM-SPSS Statistics 25.0 for Windows (IBM-SPSS Corp., Chicago, IL, USA). Associations between covariates and LLIN ownership and utilisation indicators were evaluated using the Pearson chi square (*χ*^2^) test. The odds ratio (OR) and *χ*^2^ tests were calculated by multinomial logistic regression (MNLR) for the establishment of associations or differences between the ownership/utilisation of LLINs with sociodemographic characteristics. Confounders were controlled by using independent variables from a bivariate analysis whose *χ*^2^ values were ≤0.12 in the MNLR analysis. Statistical significance was set at *p* ≤ 0.05.

## 3. Results

### 3.1. Characteristics of Study Population

From the 418 households surveyed, 2089 household residents were counted: 354 (16.9%) were children ≤ 5 years old, 704 (33.7%) were persons 6–17 years old, and 12 (0.6%) were pregnant women. Two hundred and eighty-one (67.2%) households were headed by a female and 137 (32.8%) by males. The mean age ($\bar{x}\pm \text{SD}$) of household heads was 34.3 ± 11.2 years (range 20–60) (Table 1).

SHLN = slept home last night; $\bar{x}$ = mean; SD = standard deviation.

Two hundred and sixty-four households (63.2%) were headed by married persons, 204 (48.8%) of them had acquired the primary educational level, and almost half (206, 49.3%) of the households surveyed had a family size of 3–5 persons in the household [mean ($\bar{x}\pm \text{SD}$) family size of 5.0 ± 2.5] (Table 1).

### 3.2. Sources and Ownership of LLINs

Households either purchased their LLINs or obtained them free from the second MDC antenatal clinic (ANC) or from a relation, as presented in (Table 2).

**Figure 2:**
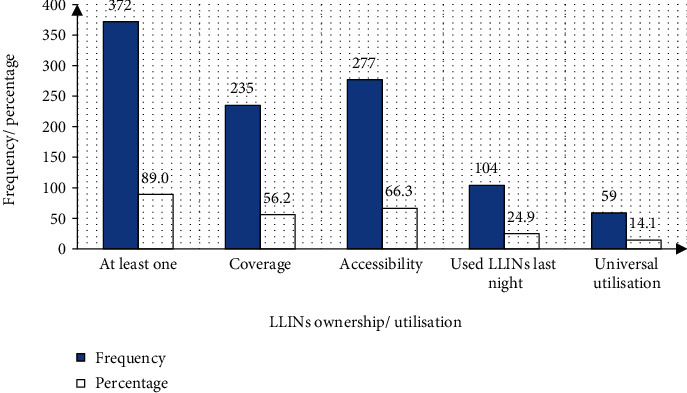
Household ownership and utilisation of LLINs.

Of the 418 households sampled, 372 (89%), 235 (56.2%), and 277 (66.3%) owned at least one LLIN, had enough LLINs (two persons per LLIN), and had household access to LLINs, respectively (Figure 2). Of the 2089 *de facto* residents covered in this study, 1862 (89.1%) lived in households with at least a bed net. A total of 985 bed nets, mean ($\bar{x}\pm \text{SD}$) density of 2.4 ± 1.5, were realized in the study. From these figures, an ownership rate of 2.6 bed nets per household and about 2 (1.9) persons per bed net was calculated in homes that owned nets. Of the 985 bed nets counted in this study, 785 (79.7%) were in use as 200 (20.3%) were reserved, reserved bed net mean ($\bar{x}\pm \text{SD}$) density of 0.5 ± 0.9 per household.

Family heads aged ≤ 20 years (*p* = 0.01; OR = 8.4; 95% C.I. 1.7–41.1) and 21–40 years (*p* = 0.09; OR = 2.2; 95% C.I. 0.9–5.7) significantly owned at least one LLIN than those aged between 41 and 60 years of age. Although households with female heads had more LLINs than those with male heads, they were significantly less likely to own a bed net compared to those headed by males (*p* = 0.04, OR = 0.5, 95% C.I. 0.2–1.0). Households with unmarried heads (Table 3) were more likely to own bed nets compared to households with married heads, but these differences were not significant (*p* = 0.32, OR = 1.4, 95% C.I. 0.7–2.7).

Households where occupants had an environmental factor (stagnant pools of water or bushes in their surroundings) were more likely to own nets compared to households with no environmental factor (*p* = 0.20; OR = 1.9; 95% C.I. 0.7–5.1) (Table 3).

### 3.3. Household Utilisation of LLINs

Of the 418 sampled households, 59 (14.1%) were those in which all the *de facto* members of the household slept under the bed net last night. One thousand and twenty-five (49.1%) of the 2089 *de facto* residents who slept home last night used LLINs. The indicators of bed net utilisation showed no association to any of the covariates. The universal utilisation of LLINs was more likely in households with female heads (OR = 1.3; 95% C.I. 0.7–2.5), most likely in households headed by those with primary education (OR = 1.6; 95% C.I. 0.6–4.1), and most likely in houses that had parts built with blocks and plank/caraboat (OR = 2.3; 95% C.I. 0.7–7.3), and those situated in environments with stagnant pools of water/surrounding bushes were less likely when compared with their counterparts.

Although with no significant association, household heads who were not married, those with primary education, those in caraboat houses, and those in the Holforth health area were more likely to have slept under LLINs the previous night when compared to their counterparts (Table 3).

### 3.4. Irregular Utilisation of LLINs

The reasons advanced for the irregular use of mosquito bed nets from respondents' perspectives were as follows: “it gives heat” (21.1%), forgetfulness (6.5%), use of fan (2.8%), difficulty to install LLINs (2.4%), and use of mosquito repellent (2.2%). Household heads acknowledged LLIN misappropriation as summarised in Table 4.

### 3.5. Association of LLIN Ownership with Utilisation

All three LLIN ownership indicators (at least a LLIN for the household, one LLIN for two persons in the household, and accessibility to LLINs in the household) had significant associations (*p* < 0.05) with the installation of LLINs on all beds in the household and the utilisation of LLINs by the entire household (universal utilisation) (Table 5).

## 4. Discussion

The Southwest Region has malaria prevalence of 46.1% [22] which constitutes one of the greatest burdens of disease in Cameroon, where malaria is highly endemic in the THD [35]. The frequency of LLIN ownership indicators owning at least a LLIN in the household, one LLIN for two persons in the household, and accessibility was 89%, 56.2%, and 66.3%, respectively, while indicators of utilisation in this study installation of LLINs on all beds in the household, SULPN, and universal utilisation were 72%, 24.9%, and 14.1%, respectively. The utilisation frequency in terms of the *de facto* members in households was 1025/2089 (49.1%).

### 4.1. Ownership of LLINs

The ownership frequency of at least one LLIN per household in this study is higher than the 47–78.8% in Fako Division [16, 23, 26, 36], the 67.1% in 2013 and 69.7% in 2017 rates reported in the Southwest Region [18], and the 59.7–73% elsewhere in Cameroon [2, 11, 24] and similar to 81.3% reported in Hohoe (Ghana) [37], 82.5% reported in Tiko (Cameroon) [23], and 89.9% in Mezam (Cameroon) [4]. The 89% ownership of at least one LLIN in our study was higher than the 41–84.1% reported elsewhere in and out of Africa [34, 38–45] and less than the 93.5% rate reported in Madagascar [46], the 98.8% in Uganda [47], and the 99.7% in Northeast Myanmar [48]. The 56.2% coverage reported in our study is more than the 36.3–47.5% reported in Fako [23] and the 28.4% in Ethiopia [40]. The variation of LLIN ownership and coverage may be accounted for by the fact that the different studies had different sample sizes and were carried out at different times, in different localities, and different study designs; some were among women of childbearing age [39], pregnant women [38], few on coverage [23, 40], while the rest were community-based studies.

### 4.2. Household Utilisation of LLINs

The proportion of household heads (24.9%) and residents (1025 (49.1%)) who slept under LLINs the previous night was small compared to ownership. Our findings were lower compared to the 50.9% among children 0–5 years in Batoke [36] and 94.1% in Fako Division, all in the Southwest Region [23]. They were also low compared to results obtained elsewhere in Cameroon such as 58.3% in rural and urban Buea [26], 69.3% in the Bamenda Health District [2], 69.7% in the Buea Health District [16], and 77.8% in Mezam Division [4] as well as out of Cameroon: 52.3% in Ethiopia [40], 75% among women of childbearing age in Nigeria [39], and 87.6% in Rwanda [38]. This low usage by the population is confirmed by other findings such as in Eastern Ethiopia with 21.5% of households [42], Mfou Health District with 42.6% [24], and Southern China with 47.2% residents [34], while 97.3% in Northeastern Myanmar [48], 80.1% in Uganda [14], and 84.2% in Madagascar [46]. The variation of LLIN utilisation may be a result of the fact that the different studies had different sample sizes and different study designs and were carried out at different times.

### 4.3. Irregular Utilisation of LLINs

Findings from this study showed that negligence or forgetfulness, heat, use of repellent or fans, and difficulty to hang up the net as well as LLIN misappropriation were accountable for low utilisation of LLINs. This has been recorded from studies in Mezam Division and the Bamenda Health District, Northwest Region of Cameroon, where LLIN usage was below the RBM rate of 80%, and nonusage was attributed to the factors similar to those outlined above (negligence, heat, and difficulty to hang up the nets) [2, 4]. These findings are similar with a survey carried out in all the ten regions of Cameroon as well as in Nigeria and Ghana whereby respondents said they used fans instead of LLINs, there were no LLINs at all, there were no mosquitoes in the locality, there was complaint of heat, and there was inconvenience of hanging up LLINs [37, 49, 50]. Our findings were different from those presented in other studies: no nets, very old and torn nets [23], poverty, insufficient nets, and colour of nets [12, 16, 24] as well as house type, locality/environment, educational level, and age [24, 39, 40]. These disparities may be a result of differences in sample sizes and study designs.

## 5. Recommendations

Pivotal to assessing ownership and utilisation rates of LLINs is obtaining epidemiological data for the communities. These findings underline the need for continuous intervention programmes to enhance LLIN distribution, installation, and most especially utilisation. Regular health education on care of the surroundings and environmental sanitation should be encouraged. This study also suggests the need for an elaborate investigation of a relationship between LLIN ownership with utilisation recorded in other health areas and their possible associations with malaria.

## 6. Strengths and Limitations

### 6.1. Strengths of the Study

Field data were obtained by well-trained field surveyors and public health personnel, who had a mastery of the Tiko Health District as they are responsible for the coding of houses during the Expanded Programme on Immunisation (EPI) and MDC campaigns. The quality of data collected was assured through the multistage sampling strategy and pretesting of questionnaires to minimize bias.

### 6.2. Limitations of the Study

This was a cross-sectional study representing the snapshot of the population within the study period and does not show cause and effect since the predictor and outcome variables were measured at the same time. Data was collected through self-reporting, and thus, there is a possibility of bias where the respondent provides socially acceptable answers. Recall bias can also affect some of the responses and subsequently the results of the study. In this study, however, respondents were required to only recall whether they and the occupants of their households slept under a LLIN the previous night, as well as the source and number of LLINs in the household.

## 7. Conclusion

This is the first study on the ownership and utilisation of LLINs in the THD of Cameroon. Although the ownership of LLINs of 89% (95% C.I. 85.63–91.65) was above the RBM-recommended standard of 80%, the utilisation rate of 49.1% was very low.

## Figures and Tables

**Table 1:** Sociodemographic characteristics of the study population.

Characteristic	Category	Health area				
		*n* (%)	Holforth	Likomba	Mondoni	Mudeka
Age groups (in years)	≤20	15 (3.6)	4 (2.4)	10 (7.6)	1 (3.2)	0 (0.0)
	21–40	295 (70.6)	121 (72.9)	111 (84.7)	22 (71.0)	41 (45.6)
	41–60	108 (25.8)	41 (24.7)	10 (7.6)	8 (25.8)	49 (54.4)
	Mean age ($\bar{x}\pm \text{SD}$)	34.3 ± 11.2	35.5 ± 10.3	27.9 ± 7.4	35.7 ± 12.3	40.7 ± 12.3

Sex	Female	281 (67.2)	126 (75.9)	80 (61.1)	23 (74.2)	52 (57.8)
	Male	137 (32.8)	40 (24.1)	51 (38.9)	8 (25.8)	38 (42.2)

Marital status	Not married	154 (36.8)	65 (39.2)	53 (40.5)	11 (35.5)	25 (27.8)
	Married	264 (63.2)	101 (60.8)	78 (59.5)	20 (64.5)	65 (72.2)

Education	Primary	204 (48.8)	72 (43.4)	64 (48.9)	16 (51.6)	52 (57.8)
	Secondary	170 (40.7)	72 (43.4)	55 (42.0)	13 (41.9)	30 (33.3)
	Tertiary	44 (10.5)	22 (13.3)	12 (9.2)	2 (6.5)	8 (8.9)

House type	Caraboat	68 (16.3)	3 (1.8)	8 (6.1)	0 (0.0)	57 (63.3)
	Caraboat/block	63 (15.1)	1 (0.6)	37 (28.2)	24 (77.4)	1 (1.1)
	Cement block	287 (68.7)	162 (97.6)	86 (65.6)	7 (22.6)	32 (35.6)

Number of bedrooms	≤2	319 (76.3)	132 (79.5)	111 (84.7)	27 (87.1)	49 (54.4)
	3–4	80 (19.1)	28 (16.9)	20 (15.3)	4 (12.9)	28 (31.1)
	≥5	19 (4.6)	6 (3.6)	0 (0.0)	0 (0.0)	13 (14.4)
	Bedroom density ($\bar{x}\pm \text{SD}$)	1.9 ± 1.2	1.7 ± 1.1	1.7 ± 0.8	1.6 ± 0.7	2.6 ± 1.6

Family size	≤2	60 (14.3)	34 (20.5)	12 (9.2)	4 (12.9)	10 (11.1)
	3–5	206 (49.3)	79 (47.6)	63 (48.1)	16 (51.6)	48 (53.3)
	6–8	120 (28.7)	42 (25.3)	48 (36.6)	10 (32.3)	20 (22.2)
	≥9	32 (7.7)	11 (6.6)	8 (6.1)	1 (3.2)	12 (13.3)
	Mean family size ($\bar{x}\pm \text{SD}$)	5.0 ± 2.5	4.6 ± 2.5	5.3 ± 2.3	5.2 ± 2.2	5.2 ± 2.7

Children 0–5 SHLN	0–2	398 (95.2)	160 (96.4)	125 (95.4)	27 (87.1)	86 (95.6)
	3–4	20 (4.8)	6 (3.6)	6 (4.6)	4 (12.9)	4 (4.4)

Net ownership	At least one LLIN	372 (89.0)	140 (84.3)	122 (93.1)	30 (96.8)	80 (88.9)
	LLIN density ($\bar{x}\pm \text{SD}$)	2.4 ± 1.6	2.2 ± 1.7	2.6 ± 1.5	2.3 ± 1.0	2.3 ± 1.6
	Total	418	166	131	31	90

**Table 2:** Sources of LLINs.

Source of LLIN	Frequency (%)
First MDC	44 (10.5)
Second MDC	315 (75.4)
Antenatal clinic (ANC)	66 (15.4)
Bought	18 (4.3)
From a relation	11 (2.6)

**Table 3:** Association of sociodemographic characteristics with LLIN ownership and utilisation.

Dependent variable →	Ownership				Utilisation			
	At least one LLIN (*n* = 372)		Coverage (*n* = 235)		Universal use (*n* = 59)		Used last night (*n* = 104)	
Independent variable ↓	*p* value	OR (95% C.I.)	*p* value	OR (95% C.I.)	*p* value	OR (95% C.I.)	*p* value	OR (95% C.I.)
Age groups (in years)								
≤20	**0.01**	8.4 (1.7–41.1)^†^	0.10	2.6 (0.8-8.3)^†^	0.82	0.8 (0.2–4.5)	0.82	0.9 (0.2–3.1)
21–40	0.09	2.2 (0.9–5.7)^†^	0.06	1.7 (1.0–2.8)^†^	0.51	0.8 (0.4–1.7)	0.90	1.0 (0.6–2.0)
41–60	Ref	1.0	Ref	1.0	Ref	1.0	Ref	1.0
Sex								
Female	**0.04**	0.5 (0.2–1.0)	0.71	1.1 (0.7–1.7)	0.37	1.3 (0.7–2.5)^†^	0.52	0.8 (0.5-1.4)
Male	Ref	1.0	Ref	1.0	Ref	1.0	Ref	1.0
Marital status								
Not married	0.32	1.4 (0.7–2.7)^†^	0.42	0.8 (0.6-1.3)	0.65	0.9 (0.5–1.6)	0.33	1.3 (0.8-2.1)
Married	Ref	1.0	Ref	1.0	Ref	1.0	Ref	1.0
Education								
Primary	0.82	1.2 (0.3–3.8)^†^	0.17	1.7 (0.8–3.6)^†^	0.30	1.6 (0.6-4.1)^†^	0.31	1.5 (0.7-3.6)^†^
Secondary	0.48	1.5 (0.5–5.0)^†^	0.28	1.5 (0.7–3.2)^†^	0.52	1.4 (0.5–3.3)^†^	0.68	0.8 (0.4-1.9)
Tertiary	Ref	1.0	Ref	1.0	Ref	1.0	Ref	1.0
House type								
Caraboat	0.66	0.8 (0.2–2.5)	0.58	0.8 (0.4-1.7)	0.79	1.2 (0.4-3.5)^†^	0.62	1.3 (0.5-3.5)^†^
Caraboat/block	0.29	0.4 (0.1–2.2)	0.50	0.8 (0.4-1.6)	0.14	2.3 (0.7–7.3)^†^	0.06	0.5 (0.2-1.0)
Cement block	Ref	1.0	Ref	1.0	Ref	1.0	Ref	1.0
Environmental risk factor^∗^								
No	0.20	1.9 (0.7–5.1)^†^	0.84	1.1 (0.5-2.4)^†^	0.60	1.4 (0.4-4.2)^†^	0.69	0.8 (0.3-2.0)
Yes	Ref	1.0	Ref	1.0	Ref	1.0	Ref	1.0
Health area^∗^								
Holforth	0.33	0.3 (0.0–3.4)	0.75	0.8 (0.3-2.5)	0.06	0.2 (0.1-1.0)	0.44	1.8 (0.4-8.1)^†^
Likomba	0.92	0.9 (0.3–2.8)	0.15	0.6 (0.3–1.2)	0.40	0.6 (0.2-1.8)	0.03	0.3 (0.1-0.9)
Mondoni	0.09	0.3 (0.1–1.2)	0.05	0.5 (0.2–1.0)	0.84	0.9 (0.3-2.8)	0.11	0.4 (0.2-1.2)
Mudeka	Ref	1.0	Ref	1.0	Ref	1.0	Ref	1.0

OR = odds ratio; C.I. = confidence interval; Ref = reference group. Boldface numbers indicate significant *p* values. ^∗^Variable with chi square *p* value < 0.05.

**Table 4:** Rationale for irregular use of LLINs.

Reasons for irregular use of LLINs	Frequency (%)
Forgot	27 (6.5)
It gives heat	88 (21.1)
Repellent was used	9 (2.2)
Used fan	12 (2.9)
Difficult to hang	10 (2.4)
*Other uses of LLINs/LLIN misappropriation*	
Window screens	74 (40.9)
Nurse huckleberry/garden	52 (28.7)
Drying of things and egussi	26 (14.4)
Fishing	15 (8.3)

**Table 5:** Association of LLIN ownership indicators with utilisation indicators.

Utilisation indicator ↓		No	Yes	Total	*χ* ^2^	*p* value
		Own at least one LLIN				
Install LLINs on all beds in HH	No	45 (97.8)	72 (19.4)	117 (28.0)	125.07	4.92 × 10^−29^
	Yes	1 (2.2)	300 (80.6)	301 (72.0)		
Universal utilisation	No	46 (100.0)	313 (81.4)	359 (85.9)	8.50	3.56 × 10^−3^
	Yes	0 (0.0)	59 (15.9)	59 (14.1)		
Slept under LLINs last night	No	31 (67.4)	283 (76.1)	314 (75.1)	1.65	0.20
	Yes	15 (32.6)	89 (23.9)	104 (24.9)		
	*Total*	46	372	418		

		Coverage				
Install LLINs on all beds in HH	No	76 (41.5)	41 (17.4)	117 (28.0)	29.61	5.30 × 10^−8^
	Yes	107 (58.5)	194 (82.6)	301 (72.0)		
Universal utilisation	No	173 (94.5)	186 (79.1)	359 (85.9)	20.09	7.38 × 10^−6^
	Yes	10 (5.5)	49 (20.9)	59 (14.1)		
Slept under LLINs last night	No	133 (72.7)	181 (77.0)	314 (75.1)	1.04	0.31
	Yes	50 (27.3)	54 (23.0)	104 (24.9)		
	*Total*	183	235	418		

		Accessibility				
Install LLINs on all beds in HH	No	68 (48.2)	49 (17.7)	117 (28.0)	43.23	4.87 × 10^−11^
	Yes	73 (51.8)	228 (82.3)	301 (72.0)		
Universal utilisation	No	130 (92.2)	229 (82.7)	359 (85.9)	7.00	8.17 × 10^−3^
	Yes	11 (7.8)	48 (17.3)	59 (14.1)		
Slept under LLINs last night	No	99 (70.2)	215 (77.6)	314 (75.1)	2.74	9.78∗10^−2^
	Yes	42 (29.8)	62 (24.4)	104 (24.9)		
	*Total*	141	277	418		

Boldface numbers indicate significant *p* values.

## Data Availability

The data used to support the findings of this study are included within the article.

## Abbreviations

HA: Health area
LLINs: Long-lasting insecticidal nets
MDC: Mass distribution campaign
OR: Odds ratio
THD: Tiko Health District
WHO: World Health Organisation.

## Supplementary Materials

Supplementary Materials S1 file: questionnaire.
