# Supplementary material for: Ownership and Utilisation of Long-Lasting Insecticidal Nets in Tiko Health District, Southwest Region, Cameroon: A Cross-Sectional Study
Source: J Parasitol Res. 2021 Feb 2;2021:8848091. doi: 10.1155/2021/8848091 (PMC7875632; doi:10.1155/2021/8848091)
Supplement: Supplementary Materials — S1 file: questionnaire. [file 8848091.f1.doc]

# APPENDIX I: HOUSEHOLD QUESTIONNAIRE ON THE OWNERSHIP AND UTILISATION OF MOSQUITO NETS IN TIKO HEALTH DISTRICT

*Please answer all the questions by completely ticking (√) in the box to the left of the answer or filling the blank spaces provided*.

Unique Identification Number (UIN): _________________

Date of Interview: _______/_______/_______ (*dd*/*mm*/*yy*)

Health Area:___________________________, Quarter: _____________________________

**SECTION A: OWNERSHIP OF MOSQUITO BED NETS**

1. a) Do you have mosquito bed net(s)? ☐ Yes, ☐ No.

b) If Yes, how many? (*Tick; √, only one*).

| ☐ None | ☐ One | ☐ Two | ☐ Three | ☐ Four | ☐ Five | ☐ More than five |
| --- | --- | --- | --- | --- | --- | --- |

1. If you have mosquito bed nets in your household, how did you acquire it (them)? (*Tick; √, all that apply*).
2. ☐ The first Mass Distribution Campaign (2011)
3. ☐ The second Mass Distribution Campaign (2015)
4. ☐ Ante-Natal Clinic
5. ☐ I bought it
6. ☐ Given to me by a Relation (*Brother, Sister, Aunt, Uncle, Parents, Friend*)
7. ☐ Others (*Specify*): ___________________________________
8. a) Did you acquire mosquito bed nets during the second Mass Distribution Campaign (2015)? ☐ Yes, ☐ No.

b) If No, Why? (*Tick; √, only one*).

1. ☐ I was not censured.
2. ☐ I was told by distributors that there was a shortage of LLINs.
3. ☐ I did not go for collection.
4. ☐ I was late for collection.
5. ☐ Others (*Specify*): _________________________________

**SECTION B: USAGE OF MOSQUITO BED NETS**

1. Are the mosquito bed net(s) hung on all the beds in each room? ☐ Yes, ☐ No.
2. How many people in the household slept under the mosquito net last night? (*Please write the number of persons, say 0, 1, 2, 3, … years old, in the box below the option*).

| Children 0 – 5 | Children 6 – 11 | Children 12 – 17 | Persons 18 and above | Expectant mother | Total |
| --- | --- | --- | --- | --- | --- |
|  |  |  |  |  |  |

1. In the past week, how often were the nets used? (*Please tick; √, in the box to the left of the option*).

| ☐ 7 nights | ☐ 5 – 6 nights | ☐ 3 – 4 nights | ☐ 1 – 2 nights | ☐ 0 nights | ☐ I don’t know |
| --- | --- | --- | --- | --- | --- |

1. If mosquito bed nets were not used every night, why not? (*Tick; √, all that apply*).

**A**. ☐ It gives heat

**B**. ☐ They were dirty

**C**. ☐ No Mosquitoes,

**D**. ☐ The Coil/ Spray/ Repellent was used

**E**. ☐ Forgot,

**F**. ☐ Hard to hang

**G**. ☐ Others (*Specify*): _______________________________

1. a) Can mosquito bed nets be washed? ☐ Yes, ☐ No, ☐ I don’t know

b) If Yes, how many times do you wash mosquito bed nets in one year? (*Tick; √, only one*)

| ☐ Have No Nets | ☐ None | ☐ Once | ☐ Twice | ☐ Thrice | ☐ More than thrice |
| --- | --- | --- | --- | --- | --- |

1. If you have mosquito net(s), how do you wash it (them)? (*Tick; √, all that apply*).
2. ☐ Never washed it
3. ☐ I use Savon
4. ☐ I use detergent (*Omo, Blu, Saba, Ariel, Mada, Ozil, Elephant*)
5. ☐ I use “coco” soap
6. Others (*Specify*): __________________________________
7. How often do you use the mosquito bed net(s)? (*Tick; √, only one*).
   1. ☐ I don’t use it
   2. ☐ During the rainy season
   3. ☐ Some of the nights
   4. ☐ Every night
8. How often do you tug the net(s) to the bed(s) when brought down in the evening? (*Tick; √, only one*).

**A**. ☐ I don’t use it **C**. ☐ Every night.

**B**. ☐ Some of the nights

1. If you have mosquito net(s), from who did you learn how to use and maintain it (them)? (*Tick; √, all that apply*).
2. ☐ I have not learned yet
3. ☐ From mass media (*Television, Radio, Newspaper, Internet*)
4. ☐ From the agents of mass distribution campaign
5. ☐ From hospital staff
6. ☐ Others (*Neighbours, Friends, Relatives*)
7. How many of the available bed nets do you not use? (*Write the number*) ____________.
8. a) Apart from using bed nets to protect from mosquito bites, are there any other uses around your quarter? ☐ Yes, ☐ No, ☐ I don’t know.

b) If Yes, which other uses? (*Tick; √, all that apply*).

1. ☐ Fishing
2. ☐ Chicken shed
3. ☐ Bathing shelter
4. ☐ Wall material
5. ☐ Wire mesh on windows
6. Do you know any other method for malaria prevention? ☐ Yes, ☐ No.
7. In your opinion, which is (are) the best way(s) to prevent malaria? (*Write in the spaces provided*).
   1. ______________________________________________________.
   2. ______________________________________________________.
8. In your household, what is the main method used to protect against malaria? (*Tick; √, all that apply*).
9. ☐ Sleep under a bednet
10. ☐ Use mosquito repellent
11. ☐ Take preventive medication
12. ☐ Spray house with insecticide
13. ☐ Keep house surroundings clean
14. ☐ Others
15. ☐ I have No Malaria Prevention Method

**SECTION C: SOCIO-DEMOGRAPHIC DATA**

1. Age (*Tick; √, only one*):

**A**. **☐** 21 – 31 Years

**B**. ☐ 32 – 42 Years

**C**. ☐ 43 – 53 Years

**D**. ☐ More than 53 Years.

1. Gender (*Tick; √, only one*): ☐ Male, ☐ Female.
2. Marital Status: (*Tick; √, only one*).

**A**. ☐ Single

**B**. ☐ Married

**C**. ☐ Widow(er)

**D**. ☐ Divorced

**E**. ☐ Separated

**F**. ☐ Cohabiting

1. What was the highest level of schooling that you completed? (*Tick; √, only one*).

**A**. ☐ No formal education

**B**. ☐ Primary education

**C**. ☐ Vocational training

**D**. ☐ Secondary/ High school education,

**E**. ☐ Tertiary education (*After high school; HND, Bachelor, Master, PhD*).

1. What is your occupation? (*Tick; √, only one*).

**A**. ☐ Civil Servant

**B**. ☐ Private Sector

**C**. ☐ CDC Worker

**D**. ☐ House Wife

**E**. ☐ Student

**F**. ☐ Business,

**G**. ☐ Farming

**H**. ☐ Fishing

**I**. ☐ Others (*Specify*): ___________

1. House type (*Tick; √, only one*): **A**. ☐ Block, **B**. ☐ Wood, **C**. ☐ Mixed (*Block with attachment*)
2. How many sleeping rooms are there in the house? (*Write the number*) ___________
3. Which of these do you have around the household? (*Tick; √, all that apply*).

**A**. ☐ Bushes/ Farm/ Garden

**B**. ☐ Standing waters

**C**. ☐ None of these.

1. How many people slept in the house last night? (*Please write the number of persons, say 0, 1, 2, 3, … years old, in the box below the option*).

| Children 0 – 5 | Children 6 – 11 | Children 12 – 17 | Persons 18 and above | Total |
| --- | --- | --- | --- | --- |
|  |  |  |  |  |

1. How often do people in the household suffer from malaria? (*Tick; √, only one*).
2. ☐ Never
3. ☐ Once in two years
4. ☐ One time a year
5. ☐ Two times a year
6. ☐ Three times a year
7. ☐ More than three times a year

Thank you very much for your Patience and Cooperation

Please return the completed questionnaire to the Investigator
